# Variations in maternal care alter corticosterone and 17beta-estradiol levels, estrous cycle and folliculogenesis and stimulate the expression of estrogen receptors alpha and beta in the ovaries of UCh rats

**DOI:** 10.1186/1477-7827-9-160

**Published:** 2011-12-22

**Authors:** João PA Amorim, Luiz GA Chuffa, Giovana R Teixeira, Leonardo O Mendes, Beatriz A Fioruci, Otávio A Martins, Wílson Mello Júnior, Janete A Anselmo-Franci, Patricia FF Pinheiro, Marcelo Martinez, Francisco E Martinez

**Affiliations:** 1 Department of Structural and Cellular Biology, Institute of Biology, Universidade Estadual de Campinas - UNICAMP, Campinas-SP 13083-863, Brazil; 2 Department of Anatomy, Bioscience Institute, UNESP - Univ. Estadual Paulista, Botucatu-SP 18618-970, Brazil; 3 Department of Morphology, Stomatology and Physiology, USP - Universidade de São Paulo, Ribeirão Preto-SP 14040-900, Brazil; 4 Department of Morphology and Pathology, UFSCar - Universidade Federal de São Carlos, São Carlos-SP 13565-905, Brazil

**Keywords:** maternal care, sex steroid receptors, corticosterone, E2, ovary

## Abstract

**Background:**

Variations in maternal care are associated with neonatal stress, hormonal disturbances and reproductive injuries during adulthood. However, the effects of these variations on sex hormones and steroid receptors during ovary development remain undetermined. This study aimed to investigate whether variations in maternal care are able to influence the hormonal profile, follicular dynamics and expression of AR, ER-alpha and ER-beta in the ovaries of UCh rat offspring.

**Methods:**

Twenty-four adult UCh rats, aged 120 days, were randomly divided into two groups (UChA and UChB) and mated. Maternal care was assessed from birth (day 0) to the 10th postnatal day (PND). In adulthood, twenty adult female rats (UChA and UChB offspring; n = 10/group), aged 120 days, were euthanized by decapitation during the morning estrus.

**Results:**

UChA females (providing high maternal care) more frequently displayed the behaviors of carrying pups, as well as licking/grooming and arched back nursing cares. Also, mothers providing high care had elevated corticosterone levels. Additionally, offspring receiving low maternal care showed the highest estrous cycle duration, increased corticosterone and 17beta-estradiol levels, overexpression of receptors ER-alpha and ER-beta, increased numbers of primordial, antral and mature follicles and accentuated granulosa cell proliferation.

**Conclusions:**

Our study suggests that low maternal care alters corticosterone and 17beta-estradiol levels, disrupting the estrous cycle and folliculogenesis and differentially regulating the expression of ER-alpha and ER-beta in the ovaries of adult rats.

## Background

In mammals, physical and psychological development depends on the relationship established between the mothers and their offspring. Any disturbance during maternal care represents an important factor affecting the regulation of hypothalamic-pituitary-adrenal axis (HPA) in addition to the pups' care [1]. HPA activation is a central physiological event that is triggered in response to stress. Deficiency in maternal care leads to neonatal injuries, which are subsequently related to disease susceptibility, hormonal imbalances, reproductive damage and social problems in adulthood [2-6].

In adult rats, maternal care includes several integrated elements relating to nutrition and pup care, and these elements appear to be spontaneously enacted by primiparous females [7,8]. After birth, essential hormones, such as prolactin, oxytocin, estrogen and corticosterone may be associated with maternal interaction [9-11], behavioral and hormonal changes stimulate the female to protect their litters [12,13]. However, once the mother-pup relationship is established, the pup's activities signal to the mother to stimulate maternal care. The major stimulus is the presence of pups that attract the attention of the mother with vocalizations, body movements and smell [14-17]. Alterations in maternal care might cause deleterious effects during development, and they seem to be detrimental to female reproduction.

Ovarian steroid hormones, such as estradiol (E2), strongly influence neural circuits that regulate sexual behavior and estrous cycle [18]. The action of E2 and androgens is mediated through estrogen receptors (ER), composed of ER-α and ER-β subunits, and androgen receptors (AR), respectively. These receptors belong to a family of steroid nuclear receptors with tissue-specific functions [19,20].

Mothers who offer low maternal care, as well as their daughters, tend to exhibit a reduced level of estrogen receptor (ERα) expression in the brain regions that regulate maternal care and the hypothalamic-pituitary-gonadal axis [21-23], but little is known about the influence of maternal care on the expression of ER-α, ER-β and AR receptors in the ovarian tissue. Interestingly, this study is the first to report the impact of maternal care on ovarian ER expression. This study also demonstrates that increases in luteinizing hormone (LH) and follicle stimulating hormone (FSH) are necessary for ovulation to occur. The preovulatory LH surge is triggered by LHRH activity, which, in turn, is dependent on increased E2 levels [24-26].

UCh rats were derived from original Wistar rats and were selected for ethanol consumption at the University of Chile over almost 60 years [27]. These ethanol-preferring rats are considered a special model for understanding of the basis of alcoholism-linked characteristics, such as those found in alcohol-related human diseases.

Despite growing evidence of the consequences of maternal care on offspring development, no study has yet evaluated the effect of maternal care on ovarian activity. Therefore, this study aimed to investigate whether variation of maternal care can alter hormonal levels and estrous-cycle duration, as well as the cell proliferation index, during folliculogenesis and expression of ER-α, ER-β and AR in the UCh rat ovary.

## Methods

### Animals

Forty-eight adult male and female UChA and UChB rats, aged 60 days (225-240 g), were obtained from the Department of Anatomy, Bioscience Institute/Campus of Botucatu, IBB/UNESP - Univ Estadual Paulista. The animals were randomly divided into two groups (n = 24/group). All animals were housed in polypropylene cages (43 cm × 30 cm ×15 cm) with laboratory-grade pine shavings as bedding and maintained under controlled temperature settings (23 ± 1°C) and lighting conditions (12-h L, 12-h D photoperiod, lights switched off at 0700 h). The animals were handled in accordance with the Ethical Principles in Animal Research adopted by the Brazilian College of Animal Experimentation (COBEA) and approved by the IBB/UNESP Ethical Committee for Animal Research, Protocol 01/08-CEEA.

After UCh rats were individually housed (aged 60 days), they were given a choice between two bottles containing either water or 10% (v/v) ethanol *ad libitum *for 15 days. After this period, 12 animals per group displaying ethanol consumption less than 1.9 g ethanol/kg BW/day (UChA strain) and higher than 2.0 g ethanol/kg BW/day (UChB strain; ranging from 4 to 5 g ethanol/kg/day) were selected for the experiment [27]. For this study, the preference ratio associated with ethanol-seeking care was approximately 50%. In addition, to ensure better efficiency and prevent against damage arising from ethanol consumption during pregnancy, the animals were maintained without access to ethanol after preference determination.

### Experimental groups

Male and female rats (120 days old) derived from UChA and UChB lineages were allowed to mate. At night, mature females were housed together with males to encourage copulation. The confirmation of mating was seen in early morning by the presence of sperm on the slides. This finding was designated as day 0 of pregnancy. The rats were monitored once per day, and near the end of pregnancy, rats were monitored twice per day in order to determine time of pup's birth. The time and date of birth was fixed as the postnatal day 0 (PND 0), and the sexing and standardization in 8 pups/litter, with proper balance between male and female being ensured in order to avoiding any interference on maternal preference, also occurred at this time. During pregnancy and lactation, the following groups were formed: UChA mothers (n = 12) and UChB mothers (n = 12).

The mothers of both UCh lineages did not receive ethanol during mating, pregnancy or lactation in order to prevent the effects of Fetal Alcohol Syndrome.

### Evaluation of maternal care

Mothers and offspring were housed in individual home-cages for evaluation of maternal care. The animals were monitored by one experimenter. Food and water were provided *ad libitum*. The maternal care was evaluated from birth (PND 0) until the 10th postnatal day (PND 10) during 60 minutes of observation four times a day. This observation amounted to 1056 h of observations. During the 60 minutes observations, each female was observed every 3 minutes for a total of 80 observations/mother/day. The observations occurred at regular times each day with three periods during the light phase (0800, 1200 and 1600 h) and one period during the dark phase (2000 h) [5,7]. The measured categories of maternal care were adopted from previous work [28-31]: carrying, licking/grooming, arched-back nursing and licking/grooming, arched-back nursing, passive nursing and contact. No contact with the pups occurred when mothers were engaged in nest building, environmental exploration, self-grooming and feeding.

### Estrous cycle analysis

At 90 days of age, the estrous cycles of female offspring were monitored by colpocytological examination (vaginal smears) every day for 21 consecutive days. Cells detaching from the vaginal epithelium were removed with a pipette (Lab Mate 0.5-10 μL, UK). Filter tips containing 10 μL 0.9% saline were discarded after the vaginal secretions had been transferred to clean slides [32]. Colpocytological examination time was set at 0900 h. Each slide was analyzed under a Zeiss Axiophot II microscope (Carl Zeiss, Germany) at 10× and 25× magnifications.

### Biological sample collection

At 120 days of age, all UChA and UChB rats in estrous were weighed and euthanized by decapitation. Blood samples were collected and stored at - 80°C for further analysis. Ovaries were dissected and weighed using analytical balance (Owalabor) and were fixed by immersion in 10% buffered formalin.

### Follicle counts

These analyses were performed using 5-μm-thick slices under light microscopy, adopting one section and discarding ten sections in sequence and finally resulting in 12 repetitions/ovary [33]. The primordial, primary, growing (more than two layers until the antral cavity appear), preantral, antral and mature follicles were each counted. All data were analyzed under a Zeiss Axiophot II microscope (Carl Zeiss, Germany) using 20× magnification for primordial and primary follicles and 10X for others.

### Immunohistochemistry for Ki-67

Sections of paraplast-embedded ovaries (5 μm) from each offspring were collected on silanized glass slides and pretreated with 2 N HCl for 30 min at 37°C. Antigen retrieval was achieved by incubating the slides with 0.1% trypsin for 15 min at 37°C. After washing, the slides were blocked with 3% hydrogen peroxide in methanol for 20 min and 3% bovine serum albumin (BSA) in PBS for 1 h at room temperature. Next, slides were incubated with monoclonal anti-Rat-Ki-67 antibody (clone MIB-5, Dako, Carpinteria, CA) at a 1:50 dilution in 1% BSA in PBS and incubated overnight at 4°C. After washing with PBS, the slides were incubated for 1 h at room temperature with biotinylated goat anti-mouse IgG antibody (Santa Cruz Biotechnology, CA) diluted 1:100 in 1% BSA in PBS. After washing, the sections were incubated with avidin-biotin-peroxidase solution (diluted 1:50) for 45 min (Elite ABC kit, Vector Laboratory, Burlingame, CA, EUA). Chromogen color development was carried out with 3,3'-diaminobenzidine tetrahydrochloride. Slides were counterstained with Harris's hematoxylin. A negative control was performed by omitting the primary antibody incubation step. The data were analyzed under a Zeiss Axiophot II microscope (Carl Zeiss, Germany). To quantitatively evaluate Ki-67-immunostained nuclei (proliferation index), the total number of positive granulosa cells in 10 randomly selected follicles was counted at 40× magnification for each follicular development stage. The results were expressed as a percentage of total cells counted (number of labeled nuclei × 100/total number of cells).

### Western blotting analysis and protein quantification

After euthanasia, the ovaries from UCh offspring were rapidly removed, and tissue samples of 50 mg were immediately frozen in liquid nitrogen and stored at -80°C. All tissues were homogenized with RIPA lysis buffer (Pierce Biotechnology, Rockford, IL, USA), using a homogenizer (IKA^® ^T10 basic Ultra, Staufen, Germany). Aliquots of 10% Triton were added to homogenates, and samples were placed on dry ice for 2 h for optimal extraction. These suspensions were centrifuged at 21,912 × g for 20 min at 4°C, and the pellet was discarded. Protein concentrations were measured by the Bradford method. Total proteins were dissolved in 1.5 × sample buffer previously described by Laemmli and used for SDS-PAGE (Bio-Rad Laboratories, Hercules, CA, USA). Equal amounts of protein (70 μg) were loaded per well onto preformed gradient gels, 4-12% acrylamide (Amersham Biosciences, Uppsala, Sweden) with a Tris-glycine running buffer system for electrophoresis (60 mA fixed during 2 h). After electrophoresis, total proteins were electro-transferred (200 mA fixed by 1 h 30 min) onto 0.2 μm nitrocellulose membranes in a Tris-glycine-methanol buffer. Prestained standards were used as molecular weight markers. Thereafter, the membranes were blocked with TBS-T solution containing 3% BSA at room temperature (RT) for 60 min and subsequently incubated at 4°C overnight with rabbit primary antibody AR anti-androgen receptor (AR); rabbit clone E115 anti-ERα; and rabbit clone 68-4 anti-ERβ (dilutions of 1:1000; 1:250; 1:500 in 1% BSA, respectively). This step was followed by washing 3 × 5 min in TBS-T solution and incubation for 2 h at RT with rabbit HRP-conjugated secondary antibodies (diluted 1:1000 in 1% BSA; Sigma, St. Louis, MO, USA). After sequential washing with TBS-T, signals were enhanced by mixing 10 mL PBS, 8 μl H_2_O_2 _and 0.02 g diaminobenzidine (DAB) as chromogen. Immunoreactive bands of each protein were obtained from blots of six rats per group using image analysis software (NIS-Elements, Advanced Research, Nikon). β-actin was used as an endogenous control, and all results were expressed as means ± SEM. Immunoblotting concentrations were represented as optical densitometry values (band intensity/β-actin ratio).

### Hormone assay

Blood samples were collected into heparinized tubes from the trunk of decapitated UCh mothers and female offspring after variation of maternal care in the postpartum period. Afterwards, plasma was obtained by centrifugation at 1,200 × g for 15 min at 4°C and stored at -20°C until assayed by radioimmunoassay (RIA). Plasma samples were assayed for FSH and LH by double-antibody RIA with specific kits provided by the National Institute of Arthritis, Diabetes, Digestive and Kidney Diseases (NIADDK, Baltimore, MD, USA). The FSH primary antibody was anti-rat FSH-S11, and the standard was FSH-RP2. The antiserum for LH was LH-S10 using RP3 as reference. The lower limit of detection for FSH and LH was 0.2 ng/mL and the intra-assay coefficients of variation were 3% and 4%, respectively. Plasma concentrations of E2 and P4 were determined using Estradiol and Progesterone Maia kits (Biochem Immunosystems, Serotec, Italy). The lower detection limit and the intra-assay coefficient of variation were, respectively, 7.5 pg/mL and 2.5% for E2 and 4.1 ng/mL and 3.7% for P4. Plasma concentrations of corticosterone were determined using specific kits provided by Sigma-Aldrich, Steinheim, Germany. Assay sensitivity was 0.023 ng/mL, and the intra-assay coefficient of variation was 4.5%.

All samples were measured in duplicate and at different dilutions when necessary. In order to prevent interassay variation, all samples were assayed in the same RIA.

### Statistical analysis

The non-parametric Mann-Whitney test was used for general maternal care comparisons. Two Way Repeated Measures ANOVA was performed to evaluate the influence of time (days) along the period of observation (based on two independent factors: time and UCh varieties). Data of follicle counts and percentages were expressed as median followed by quartiles [Q1-Q3]. Student's t-test was applied to other parameters, and the results were expressed as means ± SEM. Differences were considered significant when p < 0.05. The statistical software used was *GraphPad Instat version 4 and Sigma Plot version 11.0 *for graphic design.

## Results

### Maternal care

UChA females showed greater frequencies of care behaviors than the UChB females, including carrying pups, licking/grooming (L/G) the offspring's anogenital region, arched-back nursing with L/G and arched-back nursing alone. There was no significant difference with regard to passive nursing, although the UChA lactating females presented this care less frequently than did the UChB females. The UChA females displayed additional contact care with their offspring more frequently than did the UChB females, but this difference was not statistically significant (Table 1).

**Table 1:** Frequency of maternal behaviors during the first 10 days postpartum in UCh lactating females (n = 12/group)

Behaviors	UChA Mothers	UChB Mothers
Carrying	11.21 ± 1.83	4.97 ± 0.70**
Licking/Grooming (L/G)	1.99 ± 0.24	1.01 ± 0.08*
Arched-Back Nursing and L/G	2.30 ± 0.19	0.98 ± 0.09***
Arched-Back Nursing	8.38 ± 0.17	5.05 ± 0.40***
Passive Nursing	1.28 ± 0.27	1.50 ± 0.22
Contact with the pups	3.67 ± 0.34	3.17 ± 0.47

*p < 0.005; **p < 0.001; ***p < 0.0001. Mann-Whitney test. Values are expressed as means ± SEM

**Figure 1:**
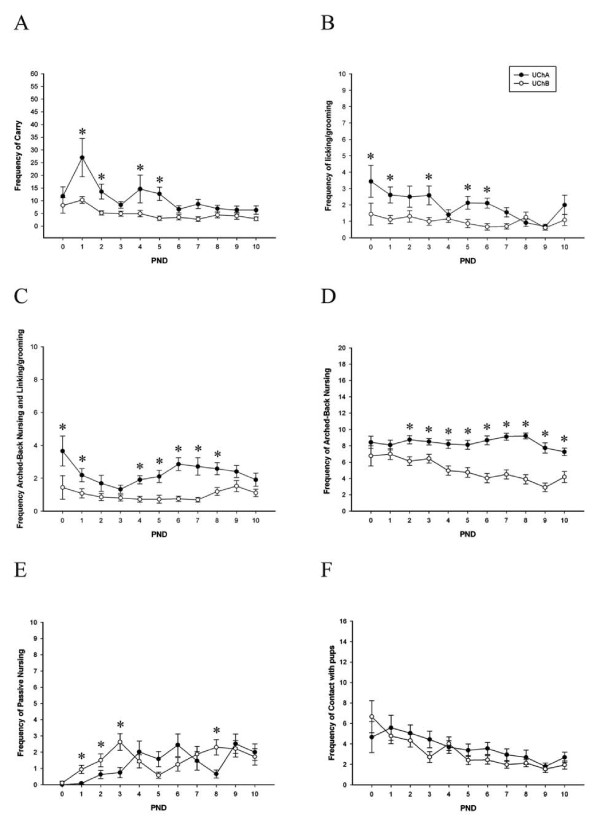
**Frequency of maternal care: low care (UChB mothers) and high care (UChA mothers)**. (A) frequency of carrying; (B) frequency of licking/grooming (L/G); (C) frequency of arched-back nursing and L/G; (D) frequency of arched-back nursing; (E) frequency of passive nursing and (F) frequency of contact with pups. Values are expressed as means ± SEM. UChA and UChB rats (n = 12/group). Two Way Repeated Measures ANOVA; *p < 0.05.

In addition, maternal care showed considerable during the first 10 days postpartum, with the highest care levels being observed at birth followed by a gradual decrease over time. Two-way repeated measures ANOVA indicated a main effect of both day (F_10,24 _= 5.1, p < 0.001) and UCh varieties (F_1,24 _= 5.8, p < 0.05) on the frequency of carrying (Figure 1A). Analysis of licking/grooming over the first 10 days postpartum indicated a main effect of both day (F_10,24 _= 2.1, p < 0.05) and UCh varieties (F_1,24 _= 15.2, p < 0.001) (Figure 1B). Analysis of arched-back nursing with L/G indicated of days (F_10,24 _= 1.6, p = 0.12) and UCh varieties (F_1,24 _= 34.8, p < 0.001) (Figure 1C). Analysis of arched-back nursing over the first 10 days postpartum indicated a main effect of both day (F_10,24 _= 2.5, p < 0.01) and varieties (F_1,24 _= 99.5, p < 0.001), and a significant interaction between day and varieties (F_10,24 _= 2.8, p < 0.01) (Figure 1D). In frequency of passive nursing the repeated measures ANOVA indicate of both days (F_10,24 _= 7.1, p < 0.001) and varieties (F_1,24 _= 4.3, p < 0.05) and a significant interaction between day and strain (F_10,24 _= 2.1, p < 0.05) (Figure 1E). Analysis of contact indicated a main effect of day (F_10,24 _= 4.3, p < 0.001) and UCh varieties (F_1,24 _= 1.0, p = 0.34), with females of all varieties decreasing their levels of contact over successive days (Figure 1F). The differences in maternal care between UChA and UChB rats were notably significant during the experiment (Figure 1).

### Analysis of the estrous cycle of female offspring

Female offspring who received low maternal care (UChB rats) exhibited the longest estrous cycle duration with prolonged metestrus stage (3-4 days arrested; Table 2).

**Table 2:** Duration of estrous cycles in the UCh female offspring (n = 10/group)

Parameters	UChA Offspring	UChB Offspring
Estrous cycle duration (days)	4.00 ± 0.10	5.86 ± 0.30*
Frequency in proestrus (days)	5.30 ± 0.15	3.63 ± 0.15*
Frequency in estrus (days)	5.50 ± 0.16	4.83 ± 0.56
Frequency in metestrus (days)	0.70 ± 0.26	3.36 ± 0.43*
Frequency in diestrus (days)	9.50 ± 0.16	9.18 ± 0.67

*p < 0.05. Student's *t*-test. Values are expressed as means ± SEM

### Ovary weight and follicles counts of female offspring

The absolute and relative ovary weights of rats receiving low maternal care (UChB) were significantly higher than those of UChA rats. Also, the UChB rat ovaries had a higher number of primordial, antral and mature follicles, while the primary and growing follicles were predominant in the ovaries of rats receiving high maternal care (Table 3).

**Table 3:** Body and ovary weights

Parameters	UChA Offspring	UChB Offspring
Body weight (g)	233.50 ± 3.16	240.0 ± 8.26*
Absolute ovary weight (g)	0.059 ± 0.001	0.074 ± 0.004*
Relative ovary weight (g/100 g)	0.025 ± 0.001	0.030 ± 0.001*
Primordial follicle	76 (59 - 113)	108 (84 - 133)*
Primary follicle	39 (14 - 70)	21.5 (15 - 27)*
Ki67 index (%)	30 (20-36)	50(34-75)*
Growth follicle	89 (49 - 106)	63.5 (46 - 73)*
Ki67 index (%)	40 (20-48)	41 (31-58)
Preantral follicle	57 (28 - 74)	53.5 (41 - 59)
Ki67 index (%)	41 (12-53)	64 (42-90)*
Antral follicle	58.5 (47 - 74)	72.5 (59 - 86)*
Ki67 index (%)	2.4 (2.1-3.5)	8.6 (7.3-9.8)*
Mature follicle	4.5 (4 - 7)	6 (2 - 11)
Ki67 index (%)	3.7 (1.6-10)	15.5 (9.09-21.87)*

Frequency and cell proliferation index of the ovarian follicles in the UCh offspring (n = 10/group)

Body weight data are expressed as means ± SEM. Values of frequency and cell proliferation index are expressed as median (Q1 - Q3). Student's *t*-test; *p < 0.05

### Plasma corticosterone and sex hormones

**Figure 2:**
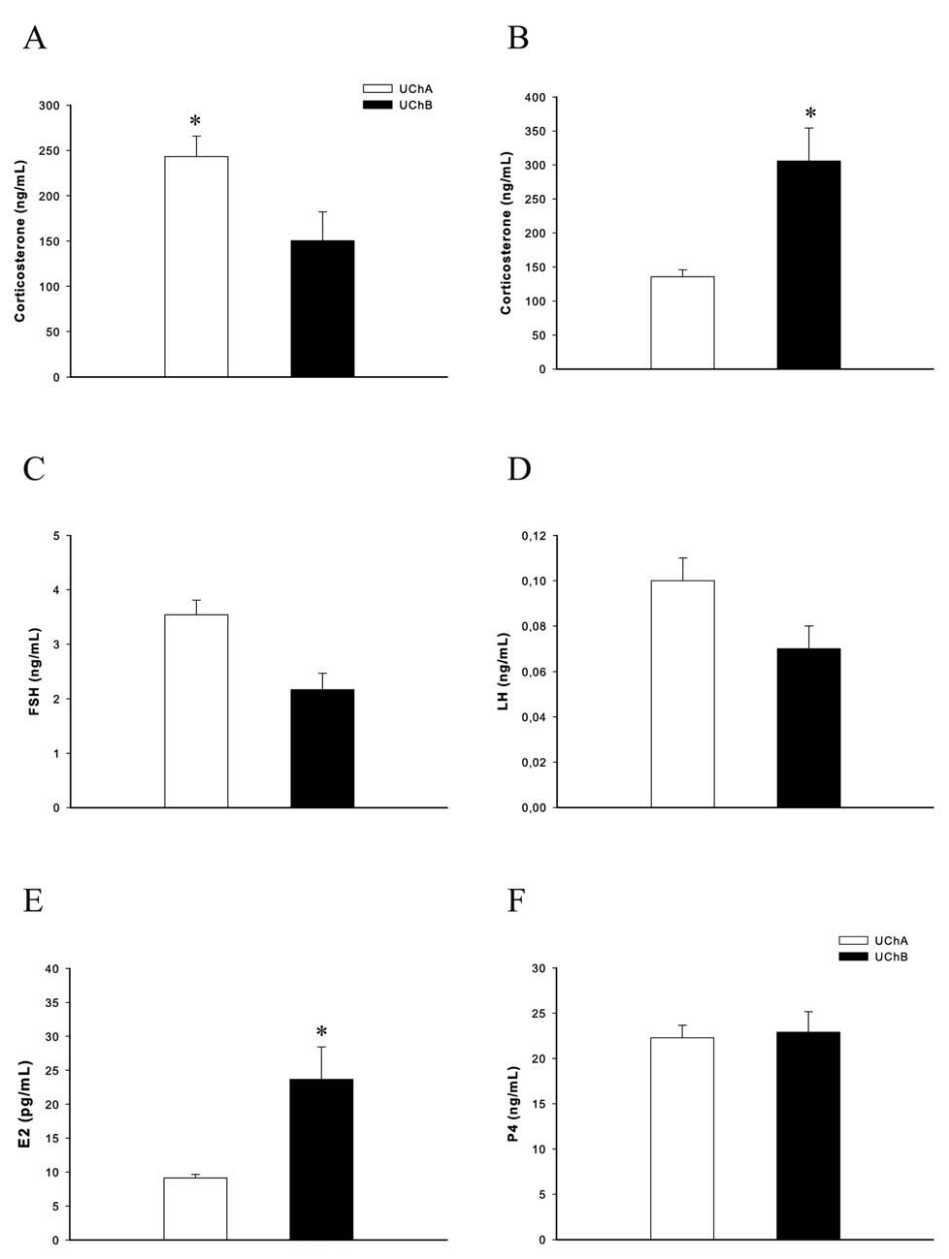
**Hormonal profile of UCh mothers and offspring after variation of maternal care in the postpartum period**. (A) Plasma corticosterone levels in the UCh mothers (ng/mL); (B) Plasma corticosterone levels in the UCh offspring (ng/mL); (C) Plasma FSH levels in the UCh offspring (ng/mL); (D) Plasma LH levels in the UCh offspring (ng/mL); (E) Plasma E2 levels in the UCh offspring (pg/mL) and (F) Plasma P4 levels in the UCh offspring (ng/mL). Values are expressed as means ± SEM. n = 10 animals/group. Student's *t*-test; * p < 0.05.

Mothers providing high maternal care (UChA rats) presented an elevation in corticosterone levels, and conversely, the low maternal care increased both corticosterone and E2 levels in the UChB offspring (Figures 2A, B, E). Plasma LH, FSH and P4 were not significantly different, due to the variations in maternal care during early postnatal life (Figures 2C, D, F).

### Analysis of ovarian AR, ER-α, ER-β in UCh offspring

**Figure 3:**
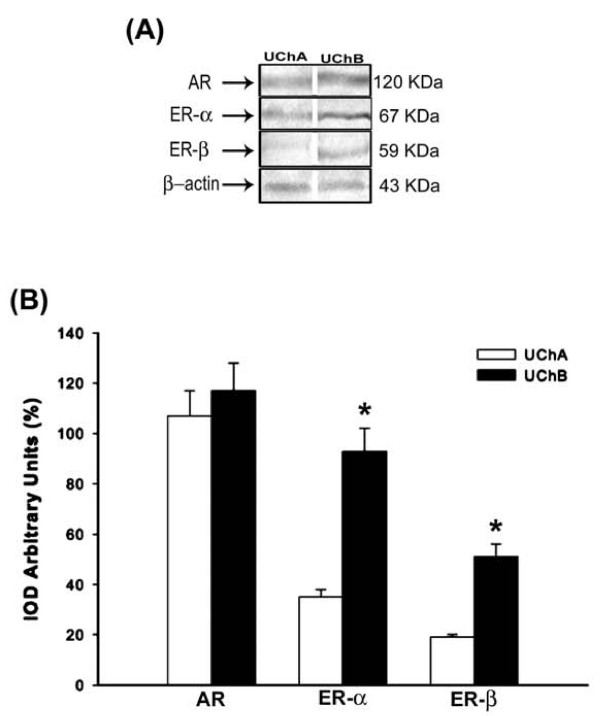
**Analysis of ovarian receptors**. (A) Representative western blotting analysis of androgen receptor (AR), estrogen receptor subunits (ER-α and ER-β) in rat ovaries after variation of maternal care. Indicated concentrations of each total protein (70 μg extracted from a pool of 6 organs/group) were used to detect specific protein expression levels in the blots (upper panel). (B) Densitometry values for AR, ER-α and ER-β levels were studied following normalization to the housekeeping gene β-actin. All results are expressed as means ± SEM (n = 6 animals/group). Student's *t*-test; * p < 0.05.

Variations in maternal care resulted in different expression patterns of ovarian sex-steroid receptors. Similarly, ovarian ER-α and ER-β were overexpressed in rats that received low maternal care, whereas AR levels did not differ between the groups (Figures 3A, B).

### Cell proliferation index (Ki-67) in UCh offspring

**Figure 4:**
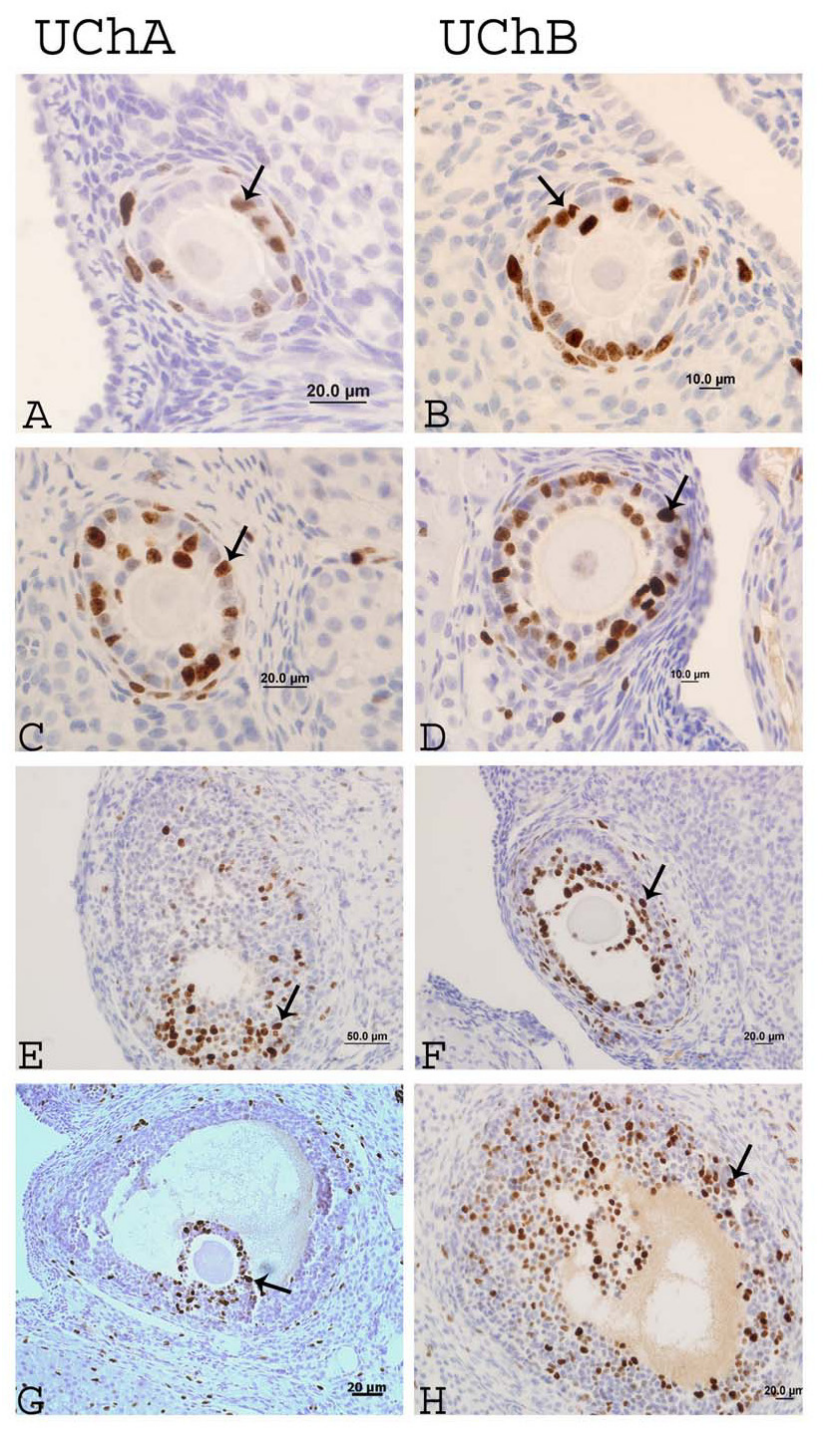
**Immunostaining for Ki-67 in different stages of ovarian follicular development in UCh offspring**. (A, B) primary follicle; (C, D) growth follicle; (E, F) preantral follicle and (G, H) antral follicle. Arrows show the positive nuclei for Ki-67.

Immunoreactivity for Ki-67 in the granulosa cells of the primary, preantral, antral and mature follicles was significantly higher in animals receiving low maternal care during early postnatal life. In contrast, growing follicles did not show significant differences between the groups (Table 3; Figure 4).

## Discussion

In the rat, variations in maternal care are associated with individual differences during the development of the neuroendocrine and reproductive system, most prominently in the females [34-36]. Evaluation of maternal care during the first 10 days postpartum revealed significant differences in maternal-infant interaction between the UCh lactating rats. UChA mothers showed higher frequencies of carrying, licking/grooming, arched-back nursing with L/G and arched-back nursing cares. However, the analysis of passive nursing and simple contact with offspring did not show significant differences. Two distinct strategies of maternal care were categorized: first, lactating UChA females offered high levels of mother-offspring contact, exhibited high infant excitement frequency (licking/grooming) and showed devotion to offspring (nutrition and heating); second, UChB mothers showed low levels of excitement and dedication to offspring and high frequencies of maternal rejection. Therefore, female UCh may be high-care (UChA mother) or low-care (UChB mother), similar to other rodent species [7,37,38].

The onset of maternal care in postpartum lactating females reflects hormone concentrations and the densities of hypothalamic receptors that stimulate and prepare the animal, beginning during the prenatal period [39]. Differently from UChB mothers, UChA mothers were more dedicated and busy during the execution of maternal care, evidencing the highest plasma corticosterone levels at the end of lactation. It is well known that mothers treated with moderate concentrations of exogenous corticosterone have increased frequency of licking/grooming and arched-back nursing care activities [40,41]. This moderate increase in maternal corticosterone is fundamental to the offspring, as it induces the appropriate development of the aminoacidergic and serotoninergic systems and maturation of hypothalamic-pituitary-adrenal axis, thereby regulating the corticosterone levels in basal conditions and under stress during adulthood [42-44]. Curiously, the UChA offspring that received high maternal care exhibited the lowest concentrations of corticosterone in adulthood. The positive effect of corticosterone on the expression of rat maternal care occurs through its action in the medial preoptic area via E2-ER activation [21]. Female rats receiving low maternal care showed high E2 concentrations, which stimulate mRNA transcription for corticotropin-releasing hormone (CRH) in the hypothalamic paraventricular nucleus (PVN) when mediated by ER-α and ER-β, thereby increasing the corticosterone secretion [45,46]. E2 also exerts a negative regulation on the neurons secreting gonadotropin-releasing hormone (GnRH). Taken together, these results point to the mutual and bidirectional interaction between the hypothalamic-pituitary-adrenal and hypothalamic-pituitary-ovarian axis [47,48].

Although FSH levels remained unchanged during the experiment, the UChB offspring had the highest E2 concentrations, as well as overexpression of their receptors. It has already been demonstrated that E2-bound ER induces multiple actions; in particular, ER-α and ER-β play different roles during ovarian activity [49]. Interestingly, E2 seems to promote its effects through activation of both ERs, mainly through ER-α in the estrus period. Our data are consistent with the previous report [50] in which ovarian ER-α was upregulated in animals that received low maternal care. To date, nuclear ER-α levels were exclusively documented in extracts from specific brain regions, being significantly elevated in the female offspring of high LG dams [21,51,36].

In UChB rats, the increase in E2 did not induce an LH surge during ovulation, arresting the estrous cycle in these rats. This result was previously described by [52] in which a prolonged estrous phase was followed by a fall in LH levels but not a lack of ovulation. Evidence suggests that the LH produced by thecal cells stimulates testosterone synthesis [53], while granulosa cells produce aromatase, an enzyme required for conversion of androgens into estrogens [54]. In our study, it is likely that androgens have been converted to estrogen because variations of stressor agents can modulate aromatase activity [55]. In addition, concentrations of plasma LH and the AR expression did not differ between the groups. Ultimately, the ovarian AR was upregulated regardless of the maternal care received. The AR responsiveness could partially explain our findings because low androgen availability leads to upregulation of its receptor. It has been proposed that androgens may act on granulosa cells throughout folliculogenesis by preventing follicular atresia and improving follicle development and maintenance of fertility [50,56]. In this regard, the AR-mediated activities are not affected during differential maternal care.

The concentrations of FSH and LH did not vary after maternal care, unlike the concentrations of E2 in UChB rats during estrus. Additionally, these rats showed higher ovarian weight and increased number of primordial, antral and mature follicles. It has recently been proposed that the androgen-AR complex is essential to promote the expression of FSH-RH during follicular growth, which stimulates the synthesis of E2 via FSH receptor activation [57]. Furthermore, androgen is responsible for stimulating early follicular growth until preantral development [58]. This regulation through E2 signaling or E2-ER binding was remarkably high in those animals receiving low maternal care, thereby contributing to follicular development. This function appears to be essentially related to primordial and primary follicles [59]. Conversely, the UChA rats exhibited a greater number of primary and growing follicles. These differences in follicular dynamics may be due to changes caused by the specific type and intensity of maternal-infant interaction beside the pattern of response mediated by activation of the HPA on the HPG axis and disturbances of the female sex hormones.

The rats that received low maternal care had higher rates of granulosa cell proliferation at most stages during follicular development. This condition is due to the mitogenic effect produced by E2, probably through an activation pathway initiated by the cyclin D2 in the granulosa cells [60]. In contrast, the UChA offspring showed a reduced granulosa cell proliferation index. It has been recently established that moderate maternal corticosterone levels during the postpartum period are responsible for attenuating cell proliferation in several tissues in adulthood [61].

## Conclusion

We conclude that low maternal care increases the plasma corticosterone and E2 levels, cell proliferation in ovarian follicles and duration of the estrous cycle and also differentially regulates the expression of ER-α and ER-β in the ovaries of the offspring during adulthood. Further studies are needed to elucidate the negative effects of low maternal care in the mother-infant interface, especially focusing on the female reproductive system.

## Competing interests

The authors declare that they have no competing interests.

## Authors' contributions

JPAA, FEM collected and analyzed the data and drafted the manuscript beyond conceiving the main idea of the study. LGAC and GRT: western blotting analysis and substantial interpretation of data. LOM, BAF, MM, OAM, WMJ and PFFP: participated in the acquisition of data, in the design of the study and in the intellectual conception of the study. JAAF participated in all RIA dosages and in interpretation of these data. All authors helped to perform the statistical analyses. All authors read and approved the final version of the manuscript.
